# Atypical Manifestation of Giardiasis: Exocrine Pancreatic Insufficiency in a Young Adult

**DOI:** 10.7759/cureus.92286

**Published:** 2025-09-14

**Authors:** Julio E Sanchez Gonzalez, Aymara Fernandez de la Vara

**Affiliations:** 1 Internal Medicine, Florida International University, Herbert Wertheim College of Medicine, Miami, USA

**Keywords:** chronic diarrhea, enteroendocrine signaling, exocrine pancreatic insufficiency, fecal elastase, giardia lamblia, giardiasis, intestinal malabsorption, pancreatic dysfunction

## Abstract

Diagnosing the underlying cause of chronic diarrhea in adults can be complex, especially when routine evaluations yield no clear etiology. Although *Giardia lamblia* is a common pathogen, its role in triggering secondary complications such as exocrine pancreatic insufficiency (EPI) is rarely recognized. Increased awareness of this association is crucial as newer diagnostic tools become more accessible in outpatient settings. We report a case of a 22-year-old male with five months of persistent watery diarrhea and abdominal discomfort following a course of clindamycin. Initial laboratory and stool studies were unrevealing, but fecal elastase-1 levels were severely reduced, consistent with EPI. The patient showed partial improvement with pancreatic enzyme replacement and cholestyramine. A multiplex polymerase chain reaction (PCR) stool panel later identified *Giardia lamblia*, and treatment with tinidazole resulted in the resolution of symptoms. This case illustrates a rare but important association between chronic giardiasis and reversible EPI. *Giardia*-induced disruption of the duodenal mucosa can impair enteroendocrine signaling and pancreatic stimulation, even in the absence of intrinsic pancreatic disease. Standard antigen testing may miss the diagnosis due to intermittent cyst shedding; multiplex molecular diagnostics offer improved sensitivity. Clinicians should maintain a broad differential in chronic diarrhea and consider early pancreatic evaluation when malabsorptive features are present. Early use of fecal elastase testing and molecular stool diagnostics in patients with persistent diarrhea can uncover overlooked but treatable conditions like *Giardia*-associated EPI. This comprehensive approach enables more accurate diagnosis, prompt treatment, and prevention of long-term nutritional and gastrointestinal complications.

## Introduction

Persistent diarrhea in young, otherwise healthy adults is a common but often diagnostically challenging complaint [1]. While infectious causes are frequently implicated, standard testing may fail to reveal an etiology, particularly in cases of chronic or relapsing symptoms [1]. Giardiasis, caused by the protozoan *Giardia lamblia*, is a leading parasitic cause of diarrheal illness worldwide, yet its chronic manifestations and extraintestinal complications, including reactive arthritis and urticaria, remain poorly recognized [2]. Emerging literature suggests a potential association between prolonged *Giardia* infection and reversible exocrine pancreatic insufficiency (EPI), a relationship that may be overlooked when diagnostic focus remains limited to either gastrointestinal pathogens or pancreatic disease alone [3]. Recognizing this interplay is essential for timely diagnosis and appropriate management, especially as multiplex molecular diagnostics and noninvasive pancreatic testing become more widely available in primary care and outpatient settings [4]. This report illustrates how integrating these tools can improve diagnostic accuracy and clinical outcomes in patients with unexplained, treatment-refractory diarrhea.

## Case presentation

A 22-year-old male with no significant past medical history presented to an outpatient primary care clinic with persistent diarrhea and abdominal pain for five months. Shortly before symptom onset, he was treated with a course of clindamycin for a piercing site infection. The patient reported that his usual bowel habits consisted of one to two daily movements, but the frequency had increased to up to six per day. He described his stools as being yellow to brown in color, with a liquid consistency. The patient also noted experiencing dull, diffuse, non-radiating abdominal pain, increased flatulence, and a strong urge to defecate within thirty minutes after meals, without any specific food triggers. In an attempt to alleviate his symptoms, the patient began using probiotics and eliminated acidic and spicy foods from his diet; however, these measures provided no relief. He denied any other significant dietary modifications in the past year, recent international travel, known food or medication allergies, or other exposure risks such as caring for children or eating at new restaurants. He had no past surgical history and was not taking any medications, vitamins, or herbal supplements. The patient also denied experiencing hematochezia, melena, bloating, nausea, vomiting, fever, chills, or any changes in weight since the onset of diarrhea.

On initial evaluation, the patient’s vital signs were temperature 36.6°C, heart rate 65 beats/minute, respiratory rate 16 breaths/minute, blood pressure 117/62 mmHg, oxygen saturation 97% on room air, and body mass index of 22.9. The physical exam showed a well-appearing, cooperative young male in no acute distress, with no signs of dehydration. The abdomen was soft, non-tender, and non-distended, with no guarding, rebound tenderness, or palpable masses. Bowel sounds were normoactive in all four quadrants, and there was no hepatosplenomegaly. The remainder of the physical exam was unremarkable. Initial laboratory studies, including complete blood count, comprehensive metabolic panel, thyroid function tests, iron studies, amylase, and lipase, were all within normal limits. Additionally, infectious and gastrointestinal workups revealed negative results for fecal leukocyte stain, fecal calprotectin, *Clostridioides difficile* toxin B, *Giardia lamblia* antigen, *Helicobacter pylori* antigen, ova and parasites, and HIV. Over the following week, the patient continued to experience persistent watery diarrhea without associated weight loss. Further tests were conducted to evaluate alternative etiologies. Serum tissue transglutaminase immunoglobulin A (IgA) and total IgA levels were both within normal limits, thereby reducing the likelihood of celiac disease. However, fecal pancreatic elastase-1 was severely decreased at <15 mcg/g (normal: >200 mcg/g). Micronutrient evaluation was performed, which showed that levels of vitamins A, E, and K were within normal limits. In contrast, vitamin B12 was decreased at 178 pg/mL (reference range: 200-900 pg/mL), and 25-hydroxyvitamin D was also reduced at 26 ng/mL (reference range: 30-100 ng/mL); therefore, supplementation was initiated.

Subsequently, the patient was evaluated by an outpatient gastroenterologist. Repeat fecal pancreatic elastase-1 testing confirmed persistently decreased levels at 48.3 mcg/g (normal: >200 mcg/g), consistent with severe pancreatic insufficiency. Treatment was initiated with pancrelipase at a dose of 40,000-126,000 units, administered as two capsules with each meal, along with cholestyramine 4 g once daily. Two weeks later, the patient reported a marked improvement in stool consistency and a decreased frequency of bowel movements; however, intermittent diffuse abdominal pain persisted. A CT scan of the abdomen and pelvis with and without contrast revealed no acute intra-abdominopelvic pathology. A Quality Diagnostics (QDx) Complete Stool Pathogen Panel (14-target multiplex polymerase chain reaction (PCR) assay) was ordered to investigate potential infectious causes and was positive for *Giardia lamblia*. The patient was prescribed tinidazole 500 mg, four tablets as a single dose, for treatment of giardiasis. At the outpatient primary care follow-up visit one week later, the patient reported complete resolution of diarrhea and overall improvement in gastrointestinal comfort, including a reduction in abdominal cramping and bloating. The patient was advised to continue monitoring symptoms and follow up as needed, with a plan for ongoing management of pancreatic enzyme replacement therapy. However, several weeks later, the patient experienced a recurrence of altered bowel habits, although less severe than prior to treatment. A colonoscopy was performed and found to be unremarkable. At the time of reporting, the gastroenterologist was planning to repeat fecal elastase testing to further evaluate the persistence of EPI following eradication of *Giardia*.

## Discussion

This case describes a rare presentation of EPI in an otherwise healthy adult following a confirmed *Giardia lamblia* infection. *Giardia lamblia* is the causative agent of giardiasis, one of the most common intestinal protozoan infections worldwide. In high-income nations, the prevalence of the condition is around 2-7%, while in low- and middle-income countries, it can reach as high as 20-30% [2]. Transmission occurs via the fecal-oral route, frequently through contaminated water sources, food, or close interpersonal contact, particularly in daycare settings with suboptimal hygiene [2]. Clinically, giardiasis spans a broad spectrum: some develop acute symptoms such as watery diarrhea, abdominal cramps, bloating, and excessive flatulence, while others exhibit chronic manifestations, including steatorrhea, weight loss, fatigue, and micronutrient deficiencies [5]. Children are especially vulnerable; chronic infections are often asymptomatic but can result in long-term effects such as growth stunting and cognitive delays [5].

**Figure 1:**
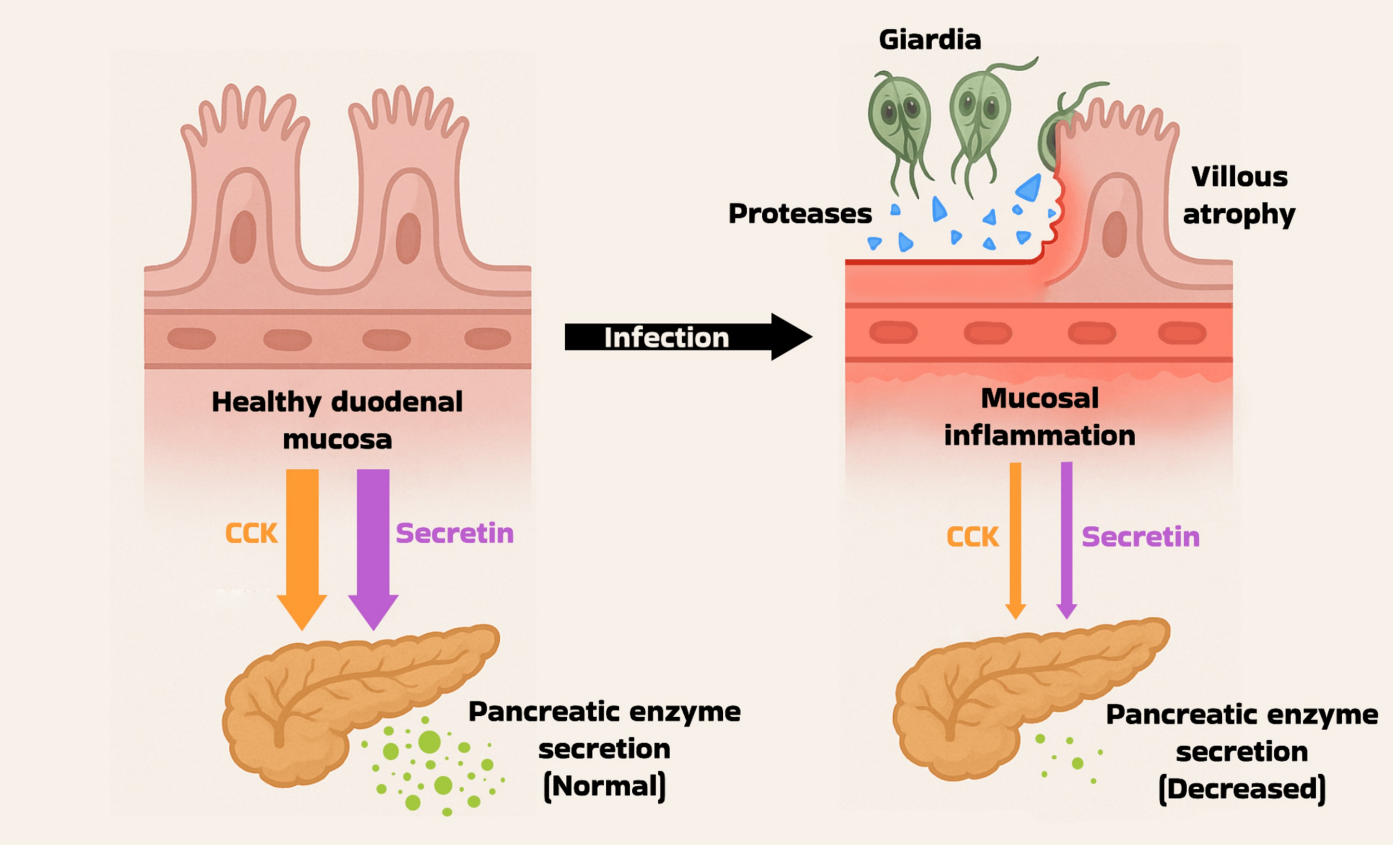
Mechanism of enteroendocrine disruption in chronic giardiasis CCK: cholecystokinin Image Credit: Julio Sanchez Gonzalez

Although *Giardia lamblia* primarily colonizes the proximal small intestine, persistent infection can profoundly disrupt the intestinal environment well beyond mechanical injury. *Giardia* trophozoites secrete proteases such as giardipain-1 that degrade tight junctions, leading to villous atrophy, increased epithelial apoptosis, and chronic duodenal inflammation [6]. These histological changes, including shortening of brush-border microvilli, crypt hyperplasia, and increased intraepithelial lymphocytic infiltration, undermine mucosal integrity, compromise brush-border enzyme activity, and impair nutrient absorption [7]. Importantly, epithelial injury reduces the release of key enteroendocrine hormones, such as cholecystokinin (CCK) and secretin, which are secreted by I-cells and S-cells in the duodenal mucosa [8]. These hormones are critical for stimulating pancreatic exocrine function; thus, their suppression can result in transient EPI in the absence of intrinsic pancreatic pathology. Figure 1 illustrates the proposed mechanism by which *Giardia lamblia* disrupts enteroendocrine signaling and contributes to EPI. Observational data in patients with celiac disease further support this mechanism, demonstrating that duodenal injury is associated with reduced pancreatic enzyme output, which improves following mucosal recovery [9]. Additionally, chronic inflammation may provoke ductal edema and intermittently obstruct ampullary outflow, further impeding enzyme delivery [10]. While the precise pathophysiological mechanisms underlying *Giardia*-associated pancreatic dysfunction remain incompletely understood, emerging reports suggest this is not an isolated phenomenon. In line with our findings, a 2021 case report documented reversible pancreatic insufficiency secondary to chronic giardiasis, emphasizing the need to consider extra-pancreatic etiologies in patients with unexplained EPI [3].

Stool microscopy and antigen assays remain first-line testing tools for *Giardia*, but intermittent cyst excretion limits their sensitivity. Studies indicate that cumulative sensitivity rises only after obtaining multiple separate stool samples [11]. In contrast, molecular methods like multiplex PCR panels, such as the QDx Complete 14-target assay, have demonstrated significantly higher sensitivity and specificity compared to single antigen tests and microscopy [12]. In our patient's case, conventional tests were repeatedly negative; only the PCR panel identified *Giardia*, enabling prompt treatment. Therefore, clinicians should consider advanced molecular diagnostics when conventional testing fails but clinical suspicion remains, particularly in protracted cases. This case highlights the importance of a broad differential when diarrhea continues despite treatment and supports early evaluation of pancreatic function when malabsorptive symptoms persist.

Metronidazole and tinidazole are considered first-line therapy against *Giardia*, with tinidazole favored in many protocols due to its single-dose convenience and comparable efficacy [13]. In this case, a single dose of tinidazole led to symptom resolution, aligning with current treatment success benchmarks. For EPI, fecal elastase measurement is a non-invasive diagnostic marker; levels below 200 mcg/g indicate insufficiency, with values under 100 mcg/g signifying severe deficiency. Pancrelipase replacement successfully restores digestive function, improving stool consistency and nutrient absorption. Additionally, bile acid sequestrants such as cholestyramine may be prescribed to manage bile acid diarrhea, a secondary mechanism frequently observed in intestinal mucosal injury, in which unabsorbed bile acids can trigger water and electrolyte secretion, leading to diarrhea [14]. In our patient, the combination of antimicrobial therapy, pancreatic enzyme replacement therapy, and cholestyramine resulted in rapid clinical improvement.

This case demonstrates the importance of a comprehensive approach to persistent diarrhea. Early evaluation of pancreatic function, such as fecal elastase testing, should be considered in patients with prolonged malabsorptive symptoms to promptly identify EPI. When initial stool studies are unrevealing, molecular diagnostics like multiplex PCR panels offer superior sensitivity and can detect overlooked infections, as demonstrated here. Effective treatment may require a multifaceted strategy addressing not only the underlying infection but also enzyme insufficiency and bile acid-induced diarrhea. Nutritional surveillance, particularly for fat-soluble vitamins, is essential to prevent long-term complications. Finally, the detection of EPI should trigger consideration of infectious or inflammatory gastrointestinal causes, not solely pancreatic imaging or endocrinology referrals. Broadening the diagnostic lens in this way can improve outcomes and avoid unnecessary delays in care.

## Conclusions

This case highlights an uncommon but clinically significant link between chronic giardiasis and secondary EPI. For clinicians, it reinforces the importance of maintaining a broad differential when evaluating persistent diarrhea, especially when symptoms are refractory to initial treatments. Incorporating early pancreatic function testing and advanced stool diagnostics, such as multiplex PCR panels, can lead to timely diagnosis and targeted therapy. Recognizing these overlapping mechanisms enables a more comprehensive, efficient approach to care that can reduce diagnostic delays, prevent nutritional deficiencies, and improve patient outcomes.
